# Pathological Complete Response Following Different Neoadjuvant Treatment Strategies for Locally Advanced Rectal Cancer: A Systematic Review and Meta-analysis

**DOI:** 10.1245/s10434-020-08615-2

**Published:** 2020-06-10

**Authors:** S. Hoendervangers, J. P. M. Burbach, M. M. Lacle, M. Koopman, W. M. U. van Grevenstein, M. P. W. Intven, H. M. Verkooijen

**Affiliations:** 1 Department of Radiation Oncology, University Medical Center Utrecht, Utrecht, The Netherlands; 2 Department of Surgery, University Medical Center Utrecht, Utrecht, The Netherlands; 3 Department of Surgery, MC Leeuwarden, Leeuwarden, The Netherlands; 4 Department of Pathology, University Medical Center Utrecht, Utrecht, The Netherlands; 5 Department of Medical Oncology, University Medical Center Utrecht, Utrecht, The Netherlands

## Abstract

**Background:**

Pathological complete response (pCR) following neoadjuvant treatment for locally advanced rectal cancer (LARC) is associated with better survival, less local recurrence, and less distant failure. Furthermore, pCR indicates that the rectum may have been preserved. This meta-analysis gives an overview of available neoadjuvant treatment strategies for LARC and analyzes how these perform in achieving pCR as compared with the standard of care.

**Methods:**

Pubmed, Embase, and Cochrane Central bibliographic databases were searched. Randomized controlled trials in which patients received neoadjuvant treatment for MRI-staged nonmetastatic resectable LARC were included. The primary outcome was pCR, defined as ypT0N0. A meta-analysis of studies comparing an intervention with standard fluoropyrimidine-based chemoradiation (CRT) was performed.

**Results:**

Of the 17 articles included in the systematic review, 11 were used for the meta-analysis. Addition of oxaliplatin to fluoropyrimidine-based CRT resulted in significantly more pCR compared with fluoropyrimidine-based CRT only (OR 1.46), but at the expense of more ≥ grade 3 toxicity. Other treatment strategies, including consolidation/induction chemotherapy and short-course radiotherapy (SCRT), did not improve pCR rates. None of the included trials reported a benefit in local control or OS. Five-year DFS was significantly worse after SCRT-delay compared with CRT (59% vs. 75.1%, HR 1.93).

**Conclusions:**

All included trials fail to deliver high-level evidence to show an improvement in pCR compared with standard fluoropyrimidine-based CRT. The addition of oxaliplatin might result in more pCR but at the expense of more toxicity. Furthermore, this benefit does not translate into less local recurrence or improved survival.

**Electronic supplementary material:**

The online version of this article (10.1245/s10434-020-08615-2) contains supplementary material, which is available to authorized users.

The aim of rectal cancer treatment is to improve survival and prevent local recurrence, while limiting treatment-related morbidity and preserving bowel, sexual, and genitourinary function.^1^^,^^2^ Consequently, patients with locally advanced rectal cancer (LARC) generally undergo neoadjuvant chemoradiation (CRT) followed by surgery.^3^^,^^4^ This combined modality approach decreases recurrence rates and improves survival compared with surgery only.^4^^,^^5^ The most frequently used neoadjuvant treatment strategy for LARC is a combination of radiotherapy (25 × 2 Gy or 28 × 1.8 Gy) and fluoropyrimidine-based chemotherapy [e.g., capecitabine or 5-fluorouracil (5FU)]. Hereby 15–20% of LARC patients achieve a pathological complete response (pCR) in which no tumor is found in the surgical resection specimen.^6^–^8^

Unfortunately, 30% of patients who receive this treatment will still die within 5 years due to local or distant recurrence.^9^ However, patients with pCR after neoadjuvant therapy are reported to have better survival, lower local recurrence, and less distant failure rates.^10^ The observation of pCR after surgery has led to a paradigm shift in rectal cancer management, in which organ preservation has become an increasingly important endpoint after neoadjuvant treatment in combination with reduction of local recurrence and survival rates.^9^ Organ-preserving treatment strategies can be considered when a complete response is detected clinically, radiographically, and/or endoscopically before surgery [i.e., clinical complete response (cCR)]. This strategy may protect patients from surgery-associated morbidity and the associated impairment in quality of life.^11^^,^^12^ As such, patients with cCR following neoadjuvant treatment are increasingly being offered watch-and-wait regimens or organ-sparing strategies, such as local excision.^13^^,^^14^ To further increase the number of eligible patients for such organ preservation strategies, physicians are searching for (new) neoadjuvant treatments with higher organ-sparing potential than the current standard of care.

Previous studies suggested that treatment intensification, i.e., adding chemotherapy or dose-escalated radiotherapy to standard chemoradiation, might enhance rectum preservation and/or improve oncological outcomes.^15^ Theoretically, intensified treatment would further downstage the tumor and any nodal disease prior to surgery and/or target potential micrometastatic disease.^4^ On the contrary, others prefer a short-course (radiation) schedule over long-course chemoradiation, based on its lower toxicity rates, better compliance, and lower cost.^16^–^19^

The present systematic review and meta-analysis gives an overview of available neoadjuvant treatment strategies for LARC and analyzes how these perform in achieving pCR (as a surrogate endpoint for cCR) compared with the current standard of care in patients with locally advanced rectal cancer based on available evidence from randomized trials.

## Methods

The present study is registered in the PROSPERO database under number CRD 42017058674.

### Search Strategy

Pubmed, Embase, and Cochrane Central bibliographic databases were searched (last update June 20, 2019) for randomized controlled trials on neoadjuvant treatment for locally advanced rectal cancer, restricted to full text and English language. The search strategy, search syntax, and characteristics of excluded studies are presented in Supplementary Tables 1 and 2 (available online). Cross-referencing was performed.

### Eligibility Criteria

Phase II–III randomized controlled trials (RCTs), conducted after the introduction of total mesorectal excision (TME) surgery in the 1980s,^20^ in which patients received neoadjuvant treatment for magnetic resonance imaging (MRI)-staged nonmetastatic LARC were included. LARC was defined as stage II–III (cT3–4N0 or T1–4N1–2) rectal cancer. All neoadjuvant treatment modalities that entailed systemic therapy and/or radiotherapy were eligible. Radiotherapy, delivered in either a short course or a long course, was considered suitable, also optionally accompanied by radiation dose escalation. Inclusion was restricted to studies using an interval of at least 4 weeks between end of neoadjuvant therapy and surgery. The primary outcome was pCR, defined as ypT0N0. Studies that did not report ypTN stage were excluded. Secondary outcomes were ≥ grade 3 toxicity [according to the Common Terminology Criteria for Adverse Events (CTCAE) version 3.0 or 4.0], surgical outcomes (complication rate and R0 resection rate), local recurrence (LR), disease free survival (DFS), and overall survival (OS). Administration of postoperative systemic therapy was not an exclusion criterion since this could not influence our primary outcome. Study selection was solely based on the primary outcome.

### Study Selection

Identified studies were listed in EndNote (1988–2012 Thomson Reuters). Two authors (S.H. and J.B.) independently screened on title and abstract. Full-text reports were retrieved and examined for eligibility criteria. Studies that only partially fulfilled the eligibility criteria were excluded. Disagreements were resolved by discussion between the two raters. Duplicates were removed, and multiple reports of the same study were linked together. Lastly, the corresponding author of each included study was contacted to obtain additional information or information at individual patient level.

### Risk of Bias Assessment

Risk of bias was assessed by the first author using the Cochrane risk-of-bias tool,^21^ including random sequence generation, allocation concealment, blinding of participants and personnel, blinding of outcome assessment, incomplete outcome data, selective reporting, and other biases. All studies were included in the analyses, irrespective of their risk of bias.

### Data Extraction

From each included trial, information about trial characteristics (study year/duration and year and country of publication), methodology (phase II or III RCT, number of arms, and sample size), characteristics of study participants [clinical tumor and nodal stage, involvement of the mesorectal fascia (MRF), and distance from the anus in cm], characteristics of intervention [agent(s), (radiotherapy) dose, duration, and interval to surgery in weeks], and outcomes [pCR (ypT0N0) rate, ≥ grade 3 toxicity (CTCAE), percentage of patients who received complete dose chemotherapy, percentage of patients that proceeded to surgery, surgical complications, R0 resection rate, and oncological outcomes (LR, DFS, and OS)] was collected. Survival data are reported as 3-year cumulative incidence rates. If available from the report, hazard ratios (HR) are also presented.

### Data Analysis

Four subgroups were created based on neoadjuvant treatment: multiagent chemoradiation (*n* = 9), induction chemotherapy (*n* = 5), consolidation chemotherapy (*n* = 2), and short-course radiotherapy and delayed surgery (SCRT-delay, *n* = 1). A systematic review of all included studies was performed. A quantitative meta-analysis on the studies that compared an intervention with standard fluoropyrimidine-based chemoradiation (25–28 × 1.8–2 Gy + capecitabine/5FU) was conducted to investigate their effect size. The Mantel–Haenszel random-effects model (REM) was applied, assuming that heterogeneity among studies was not a result of chance alone. Heterogeneity was expressed with *I*^2^.^22^ The pooled effect size was calculated from per-protocol data and is expressed as the odds ratio (OR) and its 95% confidence interval (CI).

All analyses were performed using Review Manager (RevMan), version 5.3 (The Nordic Cochrane Centre, The Cochrane Collaboration, 2014, Copenhagen, Denmark). Results were reported according the Preferred Reporting Items for Systematic Reviews and Meta-analyses (PRISMA) guidelines.^23^

## Results

### Eligible Studies

**Fig. 1:**
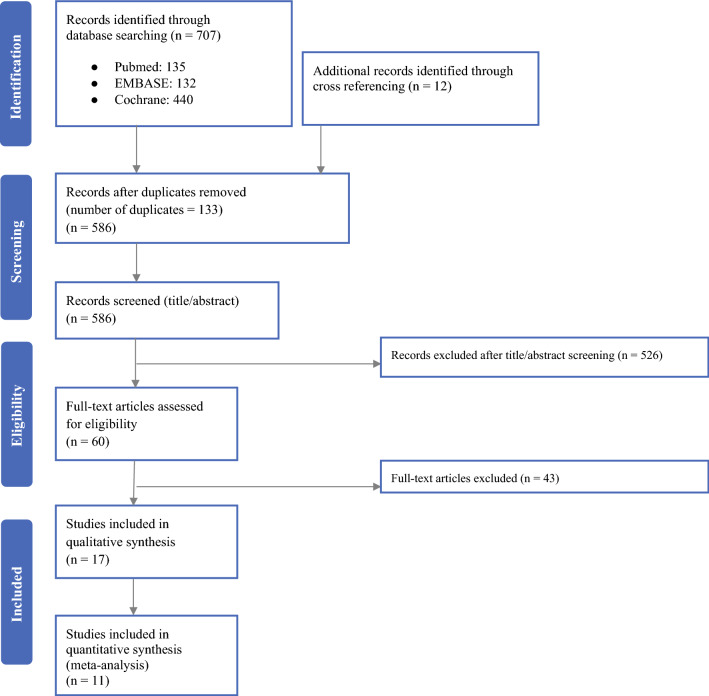
PRISMA flowchart of included studies. Reasons for exclusion provided as Supplementary Data (available online)

The literature search obtained 586 records after removal of duplicates, of which 526 records were excluded at title/abstract screening (Fig. 1). After full-text review, 17 articles met the inclusion and exclusion criteria and were included in the systematic review. Of those, 11 papers were included in the quantitative (meta)analysis. Four studies were excluded from the meta-analysis because these did not include a fluoropyrimidine-based (standard) CRT control arm. Two trials were excluded from quantitative analysis because these were the only ones in their subgroups.^19^, ^24^

### Risk of Bias

**Fig. 2:**
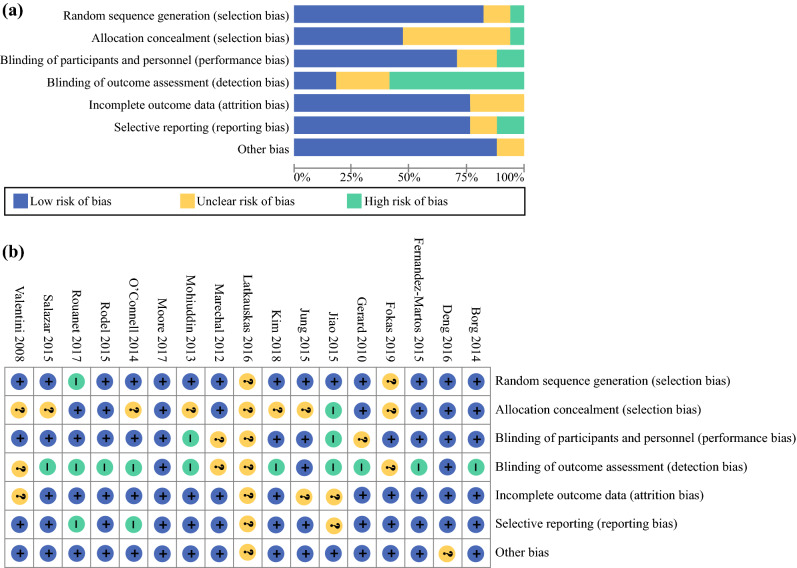
Review authors’ judgements about each risk of bias item presented as percentages across all included studies: **a** risk of bias graph and **b** risk of bias summary

In general, random sequence generation and allocation concealment were well performed and described (Fig. 2). Participants and personnel were not blinded in most studies. However, this was considered as low risk of bias since the primary outcome pCR was unlikely to be influenced by this. On the contrary, most studies lacked a blinded assessment of pCR, which could have increased the risk of detection (observer) bias.

### Characteristics of Included Studies

Ten phase II and nine phase III trials were conducted between 2001 and 2018 (Table 1). Interval to surgery varied from 4 to 12 weeks after end of neoadjuvant therapy. Detailed patient and tumor characteristics as well as an overview of administered therapy doses are presented in Supplementary Tables 3 and 4 (online accessible). The majority of patients had cT3N + tumors (Supplementary Table 3). MRF involvement was reported in eight studies and varied from 0 to 94.7%. Tumors located < 5 cm from the anus were present in 4–69.6% of included patients. The outcomes of included randomized controlled trials stratified by neoadjuvant treatment regimen are presented in Table 2.

**Table 1:** Study characteristics of randomized controlled trials stratified by neoadjuvant treatment regimen

Source			Study protocol							
Author Year Country	Study ID	Period	Study design	Tumor stage	Number of arms	Number of patients	Neoadjuvant chemotherapy	Neoadjuvant radiotherapy total dose (Gy) (number of fractions × fraction dose)	Adjuvant treatment	Interval to surgery (weeks)
*Fluoropyrimidine-based chemoradiotherapy versus multiagent chemoradiotherapy*										
Deng^19^ 2016 China	FOWARC	2010–2015	Phase III	Stage II (cT3–4N0) and stage III (cT1–4N1–2)	3	165	5FU	46–50.4 Gy (23–28 × 1.8–2)	7 cycles 5FU	4–6
						165	mFOLFOX6	46–50.4 Gy (23–28 × 1.8–2)	7 cycles mFOLFOX6	
						165	mFOLFOX6	Before or after surgery at physician discretion	6–8 cycles mFOLFOX6	
Gerard^25^ 2010 France	ACCORD 12/0405-Prodige 2	2005–2008	Phase III	cT2 in the anterior and lower rectum, cT3 or resectable cT4	2	293	Capecitabine	45 Gy (25 × 1.8)	Decision left to institution	6
						291	Capecitabine + oxaliplatin	50 Gy (25 × 2)		
Jiao^26^ 2015 China	–	2007–2010	Phase III	clinical stage II/III (cT2 in distal anterior or lower rectum, any cT3, resectable cT4, or cN1–2)	2	103	Capecitabine	50 Gy (25 × 2)	6–8 cycles FOLFOX	6–10
						103	Capecitabine + oxaliplatine	50 Gy (25 × 2)		
Jung^29^ 2015 South Korea		2009–2011	Phase II	cT3–4 or any cN	2	71	5FU	45–50.4 Gy + 4.5–9.0 Gy (25–28 × 1.8)	4 cycles 5FU	4–8
						70	Irinotecan + S-1	45–50.4 Gy + 4.5–9.0 Gy (25–28 × 1.8)		
Mohiuddin^30^ 2013 USA	RTOG-0012	2001–2003	Phase II	cT3–4	2	50	5FU	45.6 Gy + 9.6 Gy for cT3/14.4 Gy for cT4 (19 × 1.2 b.i.d.)	Recommended for patients with residual disease	4–10
						53	5FU + Irinotecan	45 Gy + 5.4 Gy for cT3/9 Gy for cT4 (25 × 1.8)		
O’Connel l^27^ 2014 USA	NSABP R-04	2004–2010	Phase III	Stage II–III (cT3–4N0 or T1–4N1–2)	4	477	5FU	45 Gy + 5.4 Gy for cT3/10.8 Gy for cT4 (25 × 1.8)	Decision left to institution	6–8
						329	5FU + oxaliplatin	45 Gy + 5.4 Gy for cT3/10.8 Gy for cT4 (25 × 1.8)		
						472	Capecitabine	45 Gy + 5.4 Gy for cT3/10.8 Gy for cT4 (25 × 1.8)		
						330	Capecitabine + oxaliplatin	45 Gy + 5.4 Gy for cT3/10.8 Gy for cT4 (25 × 1.8)		
Rodel^28^ 2015 Germany	CAO/ARO/AIO-04	2006–2010	Phase III	Any cT3–4 or cN1–2	2	623	5FU	50.4 Gy (28 × 1.8)	4 cycles 5FU	5–6
						613	5FU + oxaliplatin	50.4 Gy (28 × 1.8)	8 cycles 5FU-OX	
Valentini^60^ 2008 Italy		2002–2005	Phase II	cT3N0–2	2	83	5FU + Cisplatin	50.4 Gy (25 × 1.8 + 5.4)	Recommended for ypN + , regimen depended on physician preference	6–8
						81	Raltitrexed + oxaliplatin	50.4 Gy (25 × 1.8 + 5.4)		
Salazar^23^ 2015 Spain		2009–2011	Phase II	Stage II–III	2	46	Capecitabine	45 Gy (25 × 1.8)	Administered at the investigators’ discretion	6–8
						44	Capecitabine + bevacizumab	45 Gy (25 × 1.8)		
*Induction chemotherapy and chemoradiotherapy versus standard fluoropyrimidine-based chemoradiation*										
Borg^32^ 2014 France	INOVA	2007–2010	Phase II	cT3N0–2 in the lower rectum, cT3N0 in the midrectum or cT3N1–2	2	45	5FU + bevacizumab	45 Gy (25 × 1.8)	Left to the investigators’ discretion	6–8
						46	Induction: Bevacizumab + FOLFOX4 CRT: 5FU + bevacizumab	45 Gy (25 × 1.8)		
Fernandez-Martos^33^ 2015 Spain	GCR-3	2006–2007	Phase II	< 2 mm from MRF, ≤ 6 cm from anal verge, cT3, resectable cT4, or any cT3N+	2	52	Capecitabine + oxaliplatin	50.4 Gy (28 × 1.8)	4 cycles CAPOX	5–6
						56	Induction Capecitabine + oxaliplatin CRT: Capecitabine + oxaliplatin	50.4 Gy (28 × 1.8)	–	
Marechal^34^ 2012 Belgium			Phase II	cT2–4 N+	2	29	5FU	45 Gy (25 × 1.8)		6–8
						28	Induction: mFOLFOX6 CRT: 5FU	45 Gy (25 × 1.8)		
Rouanet^36^ 2017 France	GRECCAR-4	2011–2014	Phase II	cT3–4; CRM ≤ 1 mm, inferior tumor margin ≥ 1 cm from anal verge	4	11	FOLFIRINOX	None	Left to the investigators’ discretion. Advise: ypT0–1N0 no adjuvant treatment. ypT ≥ 2 or ypN ≥ 1: 6 cycles FOLFOX	NR
						19	Induction FOLFIRINOX CRT: Capecitabine	50 Gy (25 × 2)		
						52	Induction FOLFIRINOX CRT: Capecitabine	50 Gy (25 × 2)		
						51	Induction FOLFIRINOX CRT: Capecitabine	60 Gy (30 × 2)		
Fokas^37^ 2019 Germany	CAO/ARO/AIO-12	2015–2018	Phase II	cT3 < 6 cm from anal verge, cT3b in midrectum (≥ 6 to 12 cm), cT4, or any N+	2	156	Induction: 5FU + oxaliplatin CRT: 5FU + oxaliplatin	50.4 Gy (28 × 1.8)	Not recommended	6–12
						150	CRT: 5FU + Oxaliplatin Consolidation: 5FU + oxaliplatin	50.4 Gy (28 × 1.8)		
*Chemoradiotherapy and consolidation chemotherapy versus standard fluoropyrimidine-based chemoradiation*										
Kim^38^ 2018 South Korea	KCSG CO 14-03	2014–2016	Phase II	cT3–4	2	55	Capecitabine	50.4 Gy (28 × 1.8)	ypStage 0–1: 6 cycles CAP ypStage II–III: 6 cycles CAPOX	6–10
						53	CRT: Capecitabine Consolidation: Capecitabine + oxaliplatin	50.4 Gy (28 × 1.8)		8–10
Moore^39^ 2017 Australia	WAIT	2012–2014	Phase III	NS	2	24	5FU	45 Gy + 5.4 Gy (25 × 1.8)		10
						25	CRT: 5FU Consolidation: 5FU	45 Gy + 5.4 Gy (25 × 1.8)		
*SCRT-delay versus CRT*										
Latkauskas^24^ 2016 Lithuania		2007–2013	Phase III	Stage II–III (T3–4N0 or N +)	2	68	None	25 Gy (5 × 5)	4 cycles 5FU	6
						72	5FU	50 Gy (25 × 2)		

Underlined trials were included in the meta-analysis

*5FU* 5-fluorouracil, *AJCC* American Joint Committee on Cancer, *CAP* capecitabine, *CAPOX* capecitabine + oxaliplatin, *cN* clinical nodal stage, *CRT* chemoradiotherapy, *cT* clinical tumor stage, *FOLFOX* folinic acid + 5FU + oxaliplatin, *Gy* Gray, *MRF* mesorectal fascia, *NS* not specified, *OX* oxaliplatin, *S1* tegafur/gmieracil/oteracil

**Table 2:** Overview of outcomes of included randomized controlled trials stratified by neoadjuvant treatment regimen

Author year	Treatment summary (CT, RT, adjuvant treatment)	Included cT4 (%)	Included cN + (%)	N_CRT_	Any ≥ grade 3 CT/CRT toxicity^a^	Full CT dose	*N*_surgery_^b^	Weeks to surgery^c^	Surgical complications^d^	PCR	R0 resection	3-Year LR^e^	3-Year DFS^e^	3-Year OS^e^
*Fluoropyrimidine-based chemoradiotherapy versus multiagent chemoradiotherapy*														
Deng^19^ 2016	5FU 46–50.4 Gy Adj. 7 × 5FU	34.5	77.6	155	CTC 3.0 49 (31.6)	88.4%	141	7.6	*NR*	20 (14.2)	128 (90.8)	*NR*	*NR*	*NR*
	FOLFOX6 46–50.4 Gy Adj. 7 × FOLFOX6	33.9	81.8	158	**87 (55.1)**	94.9%	149	7.4		**41 (27.5)**	134 (89.9)			
	FOLFOX6 No RT Adj. 6–8 × FOLFOX6	30.3	72.1	163	40 (24.5)	94.5%	152	7.4		10 (6.6)	136 (89.5)			
Gerard^25^, ^61^ 2010	CAP 45 Gy Adj.: decision left to institute	5.1	70.7	293	CTC 3.0 32 (10.9)	97.2%	282	6	37 (13.1)	40 (14.2)	131^f^	6.1%	67.9%	87.6%
	CAPOX 50 Gy Adj.: decision left to institute	6.5	73	291	**74 (25.4)**	91.2%	283	6	36 (12.7)	55 (19.4)	131^f^	4.4%	72.7% HR 0.88 [0.65; 1.18]	88.3% HR 0.94 [0.59; 1.48]
Jiao^26^ 2015	CAP 50 Gy Adj. 6 − 8 × FOLFOX	37.9	77.7	103	CTC 3.0 11 (10.7)	85.4%	103	7.4	*NR*	20 (19.4)	98 (95.1)	*NR*	69.9%	86.4%
	CAPOX 50 Gy Adj. 6 − 8 × FOLFOX	34.0	78.6	103	**22 (21.4)**	81.5%	103	8		24 (23.3)	100 (97.1)		80.6%	90.3%
Jung^29^ 2015	5FU 50.4 Gy Adj. 4 × 5FU	19.7	88.7	71	CTC 4.0 0	71 (100%)	*67*	*NR*	11 (16.4)	11 (16.4)	65 (98.5)	4.5%	79.7%	*NR*
	Irinotecan-S1 45–50.4 Gy Adj. 4 × 5FU	21.4	90	70	8 (11.4)	**S-1** **90%** **Irinotecan 87.4%**	67		12 (17.9)	17 (25.4)	65 (97)	4.2%	76.6%	
Mohiuddin^30^, ^31^ 2013	5FU 45.6 Gy + 9.6/14.4 Adj.: advised for residual disease	32	38	50	CTC NS 20 (40.0)	*NR*	46	8.1	*NR*	15 (32.6)	*NR*	5-Year 16%	5-Year DSS 78% [66–90%]^g^	5-Year OS 61% [47–74%]
	5FU-Irinotecan 45 Gy + 5.4/9 Adj.: advised for residual disease	26.4	38	53	26 (49.1)		50	6.9		14 (28.0)		5-Year 17%	5-Year DSS 85% [75–95%]^g^	5-Year OS 75% [61–85%]
O’Connell^27^ 2014	5FU 45 Gy + 5.4/10.8 Adj.: decision left to institution	NR	42.1	477	CTC 4.0 129 (27.0)	*NR*	*636*	*NR*	158 (33.1)	113 (17.8) (FU/CAP)^h^	*NR*	*NR*	*NR*	*NR*
	5FU-OX 45 Gy + 5.4/10.8 Adj.: decision left to institute	NR	38.3	329	**129 (39.2)**		640		116 (35.3)	125 (19.5) (FU/CAP)^h^				
	CAP 45 Gy + 5.4/10.8 Adj.: decision left to institute	NR	42.6	472	153 (32.4)				159 (33.7)					
	CAPOX 45 Gy + 5.4/10.8 Adj.: decision left to institute	NR	38.5	330	**135 (40.9)**				125 (37.9)					
Rodel^28^ 2015	5FU 50.4 Gy Adj. 4 × 5FU	8	72.4	623	CTC 3.0 128 (20.5)	79%	615	6	272 (44.2)	81 (13.2)	584 (95.0)	4.6%	71.2% [67.6–74.9]	88.0% [85.3–90.7]
	5FU-OX 50.4 Gy Adj. 8 × 5FU-OX	6.7	73.7	613	144 (23.5)	(85%	596	6	291 (48.8)	**104 (17.4)**	567 (95.1)	2.9%	75.9% [72.4–79.5] **HR 0.79** **[0.64; 0.98]**	88.7% [86.0–91.3] HR 0.96 [0.72; 1.26]
Valentini^60^ 2008	Cisplatin-5FU 50.4 Gy Adj.: physician dependent	0	67.5	83	RTOG 6 (7.1)	*NR*	83	*NR*	15 (18.1)	18 (21.7)	*NR*	*NR*	*NR*	*NR*
	Raltitrexed-OX 50.4 Gy Adj.: physician dependent	0	63	81	13 (16.4)		81		8 (9.9)	23 (28.4)				
*Chemoradiotherapy versus chemoradiotherapy* *+* *targeted therapy*														
Salazar^23^ 2015	CAP 45 Gy Adj.: physician dependent	15.2	89.1	46	CTC 3.0 6 (13.0)	93.5%	46	7.3	*NR*	5 (10.9)	–	*NR*	*NR*	*NR*
	CAP-BEV 45 Gy Adj.: physician dependent	22.7	84.1	44	7 (16.0)	CAP 95.5% BEV 97.7%	43	7.3		7 (16.3)	–			
*Induction chemotherapy and chemoradiotherapy versus standard fluoropyrimidine-based chemoradiation*														
Borg^32^ 2014	BEV-5FU 45 Gy Adj.: physician dependent	0	82.2	45	CTC 3.0 9 (20.0)	100%	44	*NR*	15 (34.1) (≥ gr. 3)	5 (11.4)	43 (97.8)	*NR*	*NR*	*NR*
	Ind.: BEV-FOLFOX4 CRT: BEV-5FU 45 Gy Adj.: physician dependent	0	78.3	46	Overall 23 (50.0)	93.5%	42		14 (33.3)	9 (21.4)	41 (97.6)			
Fernandez-Martos^33^ 2015	CAPOX 50.4 Gy Adj. 4 × CAPOX	5.8	NR	52	CTC 3,0 15 (30.6)*	93.9%	46	*NR*	21 (45.7)	7 (15.2)	45 (97.8)	5-Year 2% [0–10.2%]	5-Year DFS 64% [49.5–75.8%]	5-Year OS 78% [63.6–87.1%]
	Ind.: CAPOX CRT: CAPOX 50.4 Gy Adj.: –	13.5	NR	56	Induction 10 (18.5) CRT 12 (22.6)	Induction 94.4% **CRT** **77.8%**	54		27 (50.0)	8 (14.8)	48 (88.9)	5-Year 5% [1.1–14.8%]	5-Year DFS 62% [48–73.4%]	5-Year OS 75% [61–84.1%]
Marechal^34^ 2012	5FU 45 Gy Adj.: –	10.3	86.2	29	CTC 3.0 2 (6.9)	97%	28	*NR*	9 (32.1)	8 (28.6)	*NR*	*NR*	*NR*	*NR*
	Ind.: FOLFOX6 CRT 5FU 45 Gy Adj.: –	7.1NR	92.9	28	**Induction** **8 (28.6)** **CRT** **2 (7.1)**	Induction 96% CRT 86%	27		7 (25.9)	7 (25.9)				
Rouanet^36^ 2017	FOLFIRINOX No RT Adj.: ypT ≥ 2/ypN ≥ 1: 6 × FOLFOX	0	81.8	11	CTC 4.0 7 (63.6)	Induction 73%	11	4.4	5 (50.0)	1 (9.1)	10 (90.9)	*NR*	*NR*	*NR*
	Ind.: FOLFIRINOX CRT: CAP 50 Gy Adj.: ypT ≥ 2/ypN ≥ 1: 6 × FOLFOX	0	73.7	19	Induction 8 (42.1) CRT 5 (26.3)	Induction 68%	19	7.6	8 (42.1)	11 (57.9)	19 (100)			
	Ind. FOLFIRINOX CRT: CAP 50 Gy Adj.: ypT ≥ 2/ypN ≥ 1: 6 × FOLFOX	23.1	96.2	52	Induction 19 (36.5) CRT 11 (21.2)	Induction 73%	52	7	16 (31.4)	7 (13.5)	43 (82.7)			
	Ind. FOLFIRINOX CRT: CAP 60 Gy Adj.: ypT ≥ 2/ypN ≥ 1: 6 × FOLFOX	25.5	98	51	Induction 8 (15.7) CRT 12 (23.5)	Induction 86%	51	7	23 (53.5)	9 (17.6)	43 (84.3)			
Fokas^37^ 2019	Ind.: 5FU-OX CRT: 5FU-OX 50.4 Gy Adj.: –	11.5	85.9	156	CTC 4.0 Induction: 34 (21.8) **CRT:** **56 (35.9)**	78%	142	6.4	59 (41.6)	27 (19.0)	130 (91.5)	*NR*	*NR*	*NR*
	CRT: 5FU-OX Cons.: 5FU-OX 50.4 Gy Adj.: –	18	90	150	CRT: 41 (27.3) Cons.: 30 (20.0)	76%	142	12.9	47 (33.1)	**38 (26.8)**	128 (90.1)			
*Chemoradiotherapy and consolidation chemotherapy versus standard fluoropyrimidine-based chemoradiation*														
Kim^38^ 2018	CAP 50.4 Gy Adj.: ypStage 0–1: 6 × CAP, ypStage II–III: 6 × CAPOX	18.2	92.7	52	CTC 4.0 Overall 2 (3.8)	*NR*	52	7.6	*NS*	3 (5.8)	52 (100)	*NR*	*NR*	*NR*
	CRT: CAP Cons.: CAPOX 50.4 Gy Adj.: ypStage 0–I: 6 × CAP, ypStage II–III: 6 × CAPOX	17	92.5	44	Overall 5 (11.4)		44	8.8		6 (13.6)	39 (88.6)			
Moore^39^ 2017	5FU 45 Gy + 5.4 Adj.: –	20.8	91.7	24	*NR*	*NR*	24	10.6	10 (41.7)	6 (25.0)	22 (91.7)	*NR*	*NR*	*NR*
	CRT: 5FU Cons.: 5FU 45 Gy + 5.4 Adj.: –	4	100	25			25	10.9	13 (52.0)	4 (16.0)	23 (92.0)			
*SCRT-delay versus CRT*														
Latkauskas^24^ 2016	No CT 25 Gy Adj.: 4 × 5FU	NR	76.5	68	*NR*	*NR*	68	6.9	24 (35.3)	3 (4.4)	57 (83.8)	3.1%	**59%** **HR 1.93** **[1.08–3.43]**	78% HR 1.64 [0.8–3.43]
	5FU 50 Gy Adj.: 4 × 5FU	NR	79.2	72			72	6.7	19 (26.8)	8 (11.1)	64 (88.9)	5.6%	**75.1%**	82.4%

Underlined trials were included in the meta-analysis. Numbers are presented as *n* (%) unless stated otherwise. Outcomes expressed in bold numbers are statistically significant

^a^CRT toxicity reported according to CTCAE 3.0 unless stated otherwise

^b^Number of participants who proceeded to surgery after neoadjuvant treatment

^c^Median interval in weeks between last radiation dose and surgery

^d^Any grade surgical complication

^e^Expressed as cumulative incidence

^f^40–45% missing data

^g^*DSS* disease-specific survival defined as death from study cancer or complications of protocol treatment

^h^CAP/5FU reported as one group with or without OX

*5FU* 5-fluorouracil, *adj*. adjuvant therapy, *APR* abdominoperineal resection, *BEV* bevacizumab, *CAP* capecitabine, *CAPOX* capecitabine + oxaliplatin, *cN* clinical nodal stage, *Cons*. consolidation chemotherapy, *CRT* chemoradiotherapy, *cT* clinical tumor stage, *CT* chemotherapy, *CTC(AE)* common terminology criteria for adverse events, *DFS* disease-free survival, *FOLFOX* folinic acid + 5FU + oxaliplatni, *Ind*. induction chemotherapy, *LR* local recurrence, *MRF* mesorectal fascia, *NR* not reported, *OS* overall survival, *OX* oxaliplatin, *pCR* pathological complete response, *RT* radiotherapy, *S1* tegafur/gimeracil/oteracil, *SCRT* short-course radiotherapy

### Fluoropyrimidine-Based CRT Versus Multiagent CRT

**Fig. 3:**
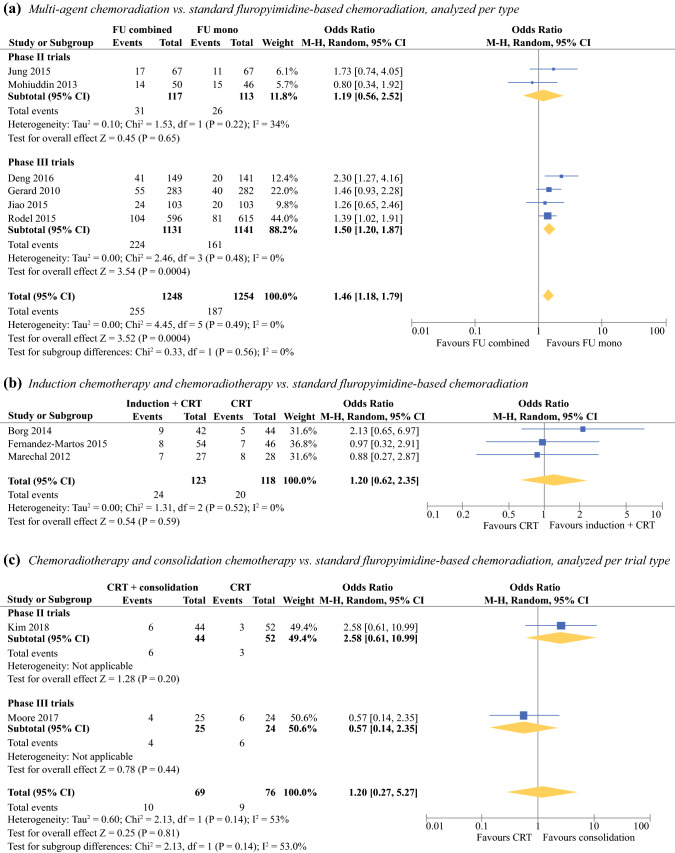
Pooled OR of pCR rates following multiagent chemoradiation, consolidation chemotherapy, and induction chemotherapy compared with standard fluoropyrimidine-based CRT

Nine trials compared fluoropyrimidine-based CRT with multiagent CRT. Six trials (two phase II trials and four phase III trials), including 2502 participants, entered the quantitative analysis. Overall, the pooled OR for pCR after multiagent CRT (*n* = 1248) versus standard CRT (*n* = 1254) was statistically significant at 1.46 (95% CI 1.18–1.79, *I*^2^ 0%). Subgroup analysis revealed that the pooled OR resulting from phase II trials was not significant (OR 1.19, 95% CI 0.56–2.52, *I*^2^ 34%), and the pooled OR from phase III trials remained statistically significant in favor of multiagent CRT (OR 1.50, 95% CI 1.20–1.87, *I*^2^ 0%, Fig. 3a).

In five trials, the experimental group received a combination of fluoropyrimidine-based chemotherapy and oxaliplatin.^25^–^29^ In patients who received fluoropyrimidine-based CRT, ≥ grade 3 toxicity occurred in 10.7–40%. In the oxaliplatin CRT group, ≥ grade 3 toxicity rates were significantly higher (21.4–49.1%), but this did not affect the number of patients that completed neoadjuvant therapy or the percentage of participants that proceeded to surgery. Neoadjuvant fluoropyrimidine-based CRT resulted in pCR in 13.2–28.3% of patients. When oxaliplatin was added to this regimen, pCR rates were 17.4–28.4%. This was statistically significant in two trials.^25^^,^^29^ No differences were seen in R0 resections or surgical complications. Two trials compared 5FU-based CRT with multiagent CRT containing irinotecan.^30^–^32^ One trial described significantly less complete dose administration in the experimental group.^30^ No differences in pCR nor in surgical and survival outcomes were seen. One trial evaluated the effect of targeted therapy (bevacizumab) added to capecitabine-based CRT in 44 patients.^24^ Compared with patients who received capecitabine-based CRT (*n* = 46), no differences were seen in toxicity or treatment compliance. All but one patient (investigational group) underwent surgery after a median interval of 7.3 weeks. pCR was achieved in 10.9% of patients in the capecitabine group and 16.3% of patients in the bevacizumab group. This difference was not statistically significant. Survival data were not available.

For all multiagent comparisons, survival and recurrence data were available from five studies.^26^^,^^27^^,^^29^–^31^ No significant differences were reported in LR or OS. Three-year cumulative incidence rates for LR and OS in the monotherapy group varied from 4.6–6.1% to 86.4–88.0%, respectively. For the multiagent group, these rates were 2.9–4.4% and 88.3–90.3%, respectively. One study reported a significant better 3-year DFS after fluoropyrimidine plus oxaliplatin-based CRT (71.2% vs. 75.9%, HR 0.79, 95% CI 0.64–0.98, Table 2).^29^

### Induction Chemotherapy

Five trials investigated the effect of CRT on pCR when this was preceded by induction chemotherapy. Induction chemotherapy plus CRT was compared with standard CRT in three phase II trials.^33^–^35^ In these trials, induction therapy consisted of multiagent chemotherapy (i.e., CAPOX or FOLFOX). Toxicity was higher after induction chemotherapy and resulted in significantly lower compliance to CRT in one trial.^34^^,^^36^ There were no differences in surgical outcomes or survival. There was no significant difference for pCR after induction chemotherapy (*n* = 123) versus standard CRT (*n* = 118) with a pooled OR of 1.20 (95% CI 0.62–2.35, *I*^2^ 0%, Fig. 3b).

Two trials (GRECCAR-4 and CAO/ARO/AIO-12) in this subgroup were not used for quantitative analysis. The GRECCAR-4 trial randomized patients based on their response to induction FOLFIRINOX.^37^ Good responders either received additional capecitabine-based CRT or underwent surgery. Poor responders were randomized to either capecitabine-based CRT or capecitabine-based CRT with dose-escalated radiotherapy (60 Gy). The trial was stopped prematurely due to low accrual rates in the good-responders arm. In the good-responder arm (*n* = 20), pCR was achieved in 1 of 11 (9.1%) patients after FOLFIRINOX alone and in 11 of 19 (57.9%) patients after induction chemotherapy with FOLFIRINOX and capecitabine-based CRT. In the poor-responder group (*n* = 103), CRT with dose-escalated radiotherapy resulted in pCR in 9 of 51 (17.6%) patients compared with 7 of 52 (13.5%) patients in the standard-CRT group. This was not a significant difference. The higher radiation dose in the poor responders arm increased R0 resection from 83 to 88%. The CAO/ARO/AIO-12 trial compared CRT and consolidation chemotherapy with CRT and induction therapy.^38^ Acute ≥ grade 3 toxicity occurred in 21.8% and 35.9% patients after induction chemotherapy alone and CRT after induction chemotherapy, respectively, compared with 27.3% in participants undergoing CRT before consolidation chemotherapy and 20% during consolidation therapy. There were no differences in number of R0 resections. pCR was significantly higher in the consolidation group. Long-term survival outcomes were not available.

### Consolidation Chemotherapy

Two RCTs (one phase II and one phase III trial) compared standard CRT with CRT followed by consolidation chemotherapy with either CAPOX or 5FU.^39^^,^^40^ Acute ≥ grade 3 toxicity was reported in one trial and did not differ between groups.^39^ R0 resections were achieved in 91.7–100% of patients after standard CRT and 88.6–92% of patients after CRT with consolidation CAPOX. This was a nonsignificant difference. The quantitative analysis for pCR in standard CRT (*n* = 76) versus CRT with consolidation CAPOX (*n* = 69) resulted in a nonsignificant difference with pooled OR of 1.17 (95% CI 0.33–4.23, *I*^2^ 54%). On the subgroup analysis, the phase II trial was in favor of CRT with consolidation therapy (OR 2.58, 95% CI 0.61–10.99),^41^ and the phase III trial was in favor of standard CRT (OR 0.57, 95% CI 0.14–2.35).^40^ None of the ORs were statistically significant (Fig. 3c). Survival data were not reported.

### Short-Course Radiotherapy and Delayed Surgery

One trial compared SCRT-delay with capecitabine-based CRT,^19^^,^^42^ resulting in a nonsignificant different pCR rate (4.4% vs. 11.1%, respectively). There were no differences in radicality or surgical complications. Five-year DFS was significantly worse after SCRT-delay compared with CRT (59% vs. 75.1%, HR 1.93, Table 2).

## Discussion

This systematic review evaluates whether pCR rates are higher following alternative neoadjuvant treatment strategies as compared with standard neoadjuvant fluoropyrimidine-based chemoradiation. All included trials fail to deliver high-level evidence to show an improvement in pathological outcomes or survival compared with standard fluoropyrimidine-based CRT. The addition of oxaliplatin to fluoropyrimidine-based CRT might result in significantly more pCR, but at the expense of more ≥ grade 3 toxicity. Furthermore, this benefit does not translate into lower rates of local recurrence or improved overall survival. Other neoadjuvant treatment strategies, including consolidation/induction chemotherapy and short-course radiotherapy with delayed surgery, were not associated with improved pCR rates. None of the included trials reported benefit in local recurrence or overall survival.

pCR following neoadjuvant therapy has been associated with improved survival^7^ and may reflect the organ-sparing potential of a treatment protocol. To increase clinical response rates after neoadjuvant treatment and herewith enable rectum preservation, different intensification strategies have been investigated in phase I–II trials, e.g., multiagent CRT, targeted therapy, radiotherapy dose-escalation, or additional chemotherapy before or after CRT [total neoadjuvant treatment (TNT)]. On multivariable metaregression, the addition of a second concurrent chemotherapy agent was not associated with improved pCR rates.^43^ In accordance with our findings, previous meta-analyses showed that the addition of oxaliplatin to preoperative chemoradiotherapy improves pCR rate, decreases LR rate, and improves DFS, but significantly worsens toxicity.^44^^,^^45^ Also, no significant difference was found in the R0 resection rate, sphincter preservation rate, permanent stoma rate, postoperative complication, mortality, or overall survival.^45^ Dose-escalated radiotherapy could be associated with higher pCR rates.^43^^,^^46^ However, this has not yet been confirmed by a randomized controlled trial and could therefore not be further investigated in the present study.^6^ TNT might manage micrometastases, increase tumor regression that enhances R0 resection rates, and increase probabilities for organ preservation.^38^ A recent meta-analysis showed that patients who received TNT followed by surgery more often achieved pCR (OR 1.39, 1.08–1.81) and better DFS (HR 0.75, 0.52–1.07) and OS (HR 0.73 (0.59–0.9) than those who received CRT only. However, this analysis was largely based on nonrandomized comparative studies, and in subgroup analyses (prospective and retrospective series), there were no statistically significant differences between TNT and CRT arms.^15^ Several trials are still ongoing,^47^^,^^48^ but to date, the superiority of TNT over standard CRT remains inconclusive.

Targeted therapy is the latest development in rectal cancer management. Translational research has led to better understanding of molecular pathways and increased the interest in targeted therapy; For example, cancer cells can express epidermal growth factor receptor (EGFR), which stimulates cell proliferation, as well as vascular endothelial growth factor receptor (VEGFR), enabling vessel formation for growth,^49^^,^^50^ and EGFR signaling might promote resistance to radiotherapy. Retrospective analyses demonstrated worse DFS and lower pCR rates in patients with rectal tumors expressing EGFR, and elevated VEGF expression in tumors has been associated with inferior survival.^49^ The addition of cetuximab, a monoclonal antibody that can sensitize cells with overexpression of EGFR to radiotherapy,^49^ has been shown not to affect the pCR rate but to significantly improve OS.^51^ Bevacizumab, an anti-VEGF antibody reducing tumor vascular density,^49^^,^ 50 did not improve pCR rates.^24^ However, these translational results are still preliminary, and clinical trials are needed.

In specific patient populations (elderly or frail) or in some countries, SCRT-delay is preferred over CRT because of its lower costs, better compliance, and less demanding nature.^52^ However, the use of SCRT remains elusive outside of Europe.^9^ Unsurprisingly, pCR rates are lower with this regimen based on its lower biological effective radiation dose compared with long-course chemoradiation. The largest randomized trial that investigated the effect of SCRT-delay was the Swedish Stockholm III trial.^53^ pCR was found in 10.4% of patients after SCRT-delay, and the risk of postoperative complications was significantly lower after SCRT-delay compared with SCRT and immediate surgery.^18^^,^^54^ However, this trial could not be included in the present study due to the lack of baseline tumor characteristics. Additionally, a combination of (induction/consolidation) chemotherapy and SCRT-delay could increase pCR rates and improve survival.^42^^,^ 55^,^^56^ The results of a large RCT on this topic are still awaited.^56^ Therefore, at this moment, SCRT-delay only seems appropriate for frail LARC patients who are unfit to undergo CRT.

This is the first systematic review to provide an overview of the most widely used and available neoadjuvant treatment modalities investigated in a randomized trial. The evaluation of pathological outcomes in relation to toxicity and surgical and survival data provides more insight in the overall effect of these regimens. Nonetheless, this meta-analysis also encountered several limitations. First, only RCTs were included, whereas a lot of new interventions are trialed in prospective single-arm phase II trials. However, these trials are prone to selection bias as well as optimism in the intervention effect and often fail to demonstrate superiority in subsequent phase III trials.^43^^,^^57^^,^ 58 Nonetheless, randomized phase II trials may also overestimate the treatment effect.^59^ We showed these differences between phase II and phase III trials in the analyses for multiagent CRT and for CRT plus consolidation chemotherapy. In addition, the RCT-limited analysis might represent a relatively well-conditioned study population,^60^ resulting in an underestimation of compliance and toxicity rates. Second, the generalizability might be limited due to strict MRI criteria and pCR definitions. Although MRI is considered to be the most optimal staging method,^2^^,^^61^ this may not be as widely available and easy accessible in all countries. In addition, the primary outcome was restricted to ypT0N0 because the interobserver agreement of other methods for tumor regression grading is low.^62^ The tumor regression grade (TRG) definition of pCR varies among approaches, and the application of a TRG is not recommended in the present TNM classification.^62^^,^ 63 Moreover, subgroups were small, and secondary outcomes could not be extracted from all included trials, which might reduce power. Third, despite strict inclusion criteria and the use of a random-effects model, uncorrected heterogeneity in study protocols might still influence the pooled effect estimates.^64^ This is for instance reflected in the different intervals between the end of neoadjuvant treatment and surgery. A prolonged interval may increase pCR rates and recurrence-free survival without compromising surgical morbidity.^65^^,^^66^ As such, higher pCR rates after consolidation therapy compared with induction therapy may be the result of an increased interval between surgery and CRT rather than the therapy itself. And lastly, only those treatments compared with a similar baseline, namely standard fluoropyrimidine-based CRT, could be used in a formal meta-analysis. The opportunity to perform an extended network meta-analysis was explored but was not reliable due to the large heterogeneity in study design and the small amount of available RCTs.

The currently available data show that there is a wide variety of neoadjuvant treatment strategies available but no high-level evidence to show an improvement in pathological outcomes and survival compared with standard of care in terms of pCR achievement and organ-sparing potential. This is probably caused by the large number of confounding factors resulting from differences in diagnosis and treatment but, more importantly, also from differences in patient and tumor characteristics. In the era of personalized treatment, more high-level evidence on tumor characteristics, (pre)treatment response prediction, long-term quality of life, and oncological outcomes after different treatment modalities is needed to support optimal and individualized rectal cancer management. This requires new, efficient, and innovative research infrastructures, such as large prospective cohorts in which trials can be conducted according to the “Trials within Cohorts” (TwiCs) design.^67^^,^^68^ This enables investigation of novel prognostic and predictive factors in large populations as well as in small subgroups of patients and simultaneously provides the platform to conduct (partly) overlapping randomized trials with robust and validated analysis methods that provide clinically relevant answers that can be directly translated into changes for routine care.^69^

## Electronic supplementary material

Below is the link to the electronic supplementary material.

Supplementary material 1 (DOCX 93 kb)
