# Supplementary material for: Pathological Complete Response Following Different Neoadjuvant Treatment Strategies for Locally Advanced Rectal Cancer: A Systematic Review and Meta-analysis
Source: Ann Surg Oncol. 2020 Jun 10;27(11):4319–36. doi: 10.1245/s10434-020-08615-2 (PMC7497700; doi:10.1245/s10434-020-08615-2)
Supplement: Supplementary file 1 — Supplementary material 1 (DOCX 93 kb) [file 10434_2020_8615_MOESM1_ESM.docx]

# SUPPLEMENTARY DATA

**Supplementary Table 1.** Search syntax (last update June 20, 2019)

| **Domain** | Locally advanced rectal cancer patients |
| --- | --- |
| **Determinant** | Neoadjuvant treatment |
| **Outcome** | Pathological complete response |
| **Pubmed / MEDLINE**  N = 135 | #1: (((((pathological complete response[Title/Abstract]) OR pCR[Title/Abstract]) OR Complete pathological response[Title/Abstract]) OR Complete response[Title/Abstract]) OR Pathological response[Title/Abstract]) OR response[Title/Abstract]  #2: ((((Locally advanced rectal cancer[Title/Abstract]) OR Advanced rectal cancer[Title/Abstract]) OR Carcinoma rectum[Title/Abstract]) OR Rectal Neoplasms [MeSH]) OR Rectal cancer[Title/Abstract]  #3: ((((((Neoadjuvant treatment[Title/Abstract]) OR Neoadjuvant therapy[Title/Abstract]) OR Neo-adjuvant treatment[Title/Abstract]) OR Neo-adjuvant therapy[Title/Abstract]) OR Preoperative treatment[Title/Abstract]) OR Preoperative therapy[Title/Abstract]) OR Neoadjuvant therapy [MeSH]  #4: ((((((RCT[Title/Abstract]) OR Randomized controlled trial[Title/Abstract]) OR Randomised controlled trial[Title/Abstract]) OR Randomized trial[Title/Abstract]) OR Randomised trial[Title/Abstract]) OR controlled trial[Title/Abstract]) OR Clinical Trial [MeSH]  #5: (((Radiotherapy[Title/Abstract]) OR Irradiation[Title/Abstract]) OR Radiation[Title/Abstract]) OR Radiotherapy [MeSH]  #6: (((chemoradiation[Title/Abstract]) OR chemoradiotherapy[Title/Abstract]) OR chemoradiotherapy[MeSH Terms]) OR combined modality therapy[MeSH Terms]  #7: ((((((Chemotherapy[Title/Abstract]) OR Chemotherapy [MeSH]) OR Consolidation chemotherapy[Title/Abstract]) OR Induction chemotherapy[Title/Abstract]) OR Consolidation chemotherapy [MeSH]) OR Induction chemotherapy [MeSH]) OR Concomitant chemotherapy[Title/Abstract]  #8: (Brachytherapy[Title/Abstract]) OR Brachytherapy [MeSH]  #9: (((#5) OR #6) OR #7) OR #8  #10: (#3) AND #9  #11: ((#1) AND #2) AND #10  #12: (#11) AND #4  NB. Radiotherapy [MeSH Terms] also incorparates “brachytherapy [MeSH Terms]” |
| **EMBASE**  N = 132 | #1: 'pathological complete response':ab,ti OR 'pcr':ab,ti OR 'complete pathological respons':ab,ti OR 'complete response':ab,ti OR 'pathological response':ab,ti OR 'response':ab,ti  #2: 'locally advanced rectal cancer':ab,ti OR 'advanced rectal cancer':ab,ti OR 'carcinoma rectum':ab,ti OR 'rectal cancer':ab,ti OR 'larc':ab,ti  #3: 'neoadjuvant':ab,ti OR 'neo-adjuvant':ab,ti OR ‘preoperative treatment’:ab,ti  #4: 'radiotherapy':ab,ti OR 'irradiation':ab,ti OR 'radiation':ab,ti OR 'brachytherapy':ab,ti  #5: 'chemoradiotherapy':ab,ti OR 'chemoradiation':ab,ti  #6: 'chemotherapy':ab,ti OR 'consolidation chemotherapy':ab,ti OR 'concurrent chemotherapy':ab,ti OR 'induction chemotherapy':ab,ti OR 'concomitant chemotherapy':ab,ti  #7: ‘brachytherapy’:ab,ti  #8: ‘RCT’:ab,ti OR ‘Randomized controlled trial’:ab,ti OR ‘Randomised controlled trial’:ab,ti OR ‘Randomized trial’:ab,ti OR ‘Randomised trial’:ab,ti OR ‘controlled trial’:ab,ti  #9: #1 AND #2  #10: #4 OR #5 OR #6 OR #7  #11: #3 AND #10  #12: #9 AND #11  # 13: #8 AND # 12 |
| **Cochrane**  N = 440 | #1  (MeSH descriptor: [Rectal Neoplasms] explode all trees  #2  'locally advanced rectal cancer':ab,ti or 'advanced rectal cancer':ab,ti or 'carcinoma rectum':ab,ti or 'rectal cancer':ab,ti or 'larc':ab,ti  #3  #1 OR #2  #4 'pathological complete response':ab,ti or 'pcr':ab,ti or 'complete pathological respons':ab,ti or 'complete response':ab,ti or 'pathological response':ab,ti or 'response':ab,ti  #5  MeSH descriptor: [Neoadjuvant Therapy] explode all trees  #6  'neoadjuvant':ab,ti OR 'neo-adjuvant':ab,ti OR ‘preoperative treatment’:ab,ti  #7  **#5 OR #6**  #8 MeSH descriptor: [Radiotherapy] explode all trees  #9  'radiotherapy':ab,ti OR 'irradiation':ab,ti OR 'radiation':ab,ti OR 'brachytherapy':ab,ti  #10  #8 OR #9  #11  MeSH descriptor: [Drug Therapy] explode all trees  #12  'chemotherapy':ab,ti OR 'consolidation chemotherapy':ab,ti OR 'concurrent chemotherapy':ab,ti OR 'induction chemotherapy':ab,ti  #13  #11 OR #12  #14  MeSH descriptor: [Chemoradiotherapy] explode all trees  #15  'chemoradiotherapy':ab,ti OR 'chemoradiation':ab,ti  #16  #14 OR #15  #17: #3 AND #4  #18: #10 OR #13 OR #16  #19: #7 AND #18  #20: #17 AND #19 |

**Supplementary Table 2.** Characteristics of excluded studies.

Reasons for exclusion in title/abstract screening:

•  Duplicate (n = 133)

•  Unrelated research question (n = 155)

•  Conference abstract/paper (n = 133)

•  Different study design (n = 108)

•  Study protocol (n = 108)

•  Abstract unavailable (n = 1)

•  English abstract/text unavailable (n = 4)

•  Different tumor site or non-adenocarcinoma (n = 5)

•  Not MRI staged (n = 4)

•  pCR not defined as ypT0N0 or not reported (n = 1)

•  Non-LARC (n = 2)

•  Irresectable or recurrent rectal cancer (n = 3)

•  Adjuvant therapy (n = 2)

Reasons for exclusion in full-text assessment:

•  Multiple reports on same study (n = 10)

•  Unrelated research question (n = 4)

•  Conference abstract/paper (n = 1)

•  Different study design (n = 2)

•  Study protocol (n = 1)

•  Full text unavailable/not accessible (n = 1)

•  Not MRI staged (n = 10)

•  pCR not defined as ypT0N0 or not reported (n = 10)

•  Non-LARC (n = 2)

**Supplementary Table 3.** Patient characteristics of included randomized controlled trials, stratified by neoadjuvant treatment regimen. Underlined trials were included in the meta-analysis. Numbers are presented as n(%), unless stated otherwise.

| **Source** | | **Treatment characteristics** | | **Tumor characteristics** | | | | | | |
| --- | --- | --- | --- | --- | --- | --- | --- | --- | --- | --- |
| **Author** | **Year** | **Neoadjuvant treatment** | **Number of patients** | **cT2** | **cT3** | **cT4** | **cN0** | **cN+** | **MRF +** | **<5cm from anal verge** |
| **Monotherapy vs. combined treatment: fluoropyrimidine-based chemoradiotherapy vs. fluoropyrimidine-based chemoradiotherapy + platinum-based chemotherapy** | | | | | | | | | | |
| **Deng** [^25^](#_ENREF_25) | 2016 | 5FU | 165 | 8 (4.8) | 100 (60.6) | 57 (34.5) | 37 (22.4) | 128 (77.6) | *NR* | 90 (54.5) |
|  |  | mFOLFOX6 | 165 | 3 (1.8) | 106 (64.2) | 56 (33.9) | 30 (18.2) | 135 (81.8) |  | 83 (50.3) |
|  |  | mFOLFOX6 | 165 | 1 (0.6) | 114 (69.1) | 50 (30.3) | 46 (27.9) | 119 (72.1) |  | 70 (42.4) |
| **Gerard** [^26^](#_ENREF_26) | 2010 | CAP | 293 | 23 (7.9) | 255 (87.0) | 15 (5.1) | 85 (29.3) | 205 (70.7) | 27 (19.6) | 204 (69.6)  (<6cm) |
|  |  | CAPOX | 291 | 21 (7.2) | 254 (87.3) | 16 (6.5) | 78 (27.0) | 211 (73.0) | 30 (19.7) | 184 (63.2)  (<6cm) |
| **Jiao** [^27^](#_ENREF_27) | 2015 | CAP | 103 | 3 (2.9) | 61 (59.2) | 39 (37.9) | 23 (22.3) | 80 (77.7) | *NR* | 25 (24.3)  (<4cm) |
|  |  | CAPOX | 103 | 2 (1.9) | 66 (64.1) | 35 (34.0) | 22 (21.4) | 81 (78.6) |  | 24 (23.3)  (<4 cm) |
| **Jung** [^30^](#_ENREF_30) | 2015 | 5FU | 71 | 0 | 57 (80.3) | 14 (19.7) | 8 (11.3) | 63 (88.7) | *NR* | 17 (23.9) |
|  |  | Irinotecan-S1 | 70 | 0 | 55 (78.6) | 15 (21.4) | 7 (10.0) | 63 (90.0) |  | 18 (25.7) |
| **Mohiuddin** [^31^](#_ENREF_31) | 2013 | 5FU | 50 | 0 | 34 (68) | 16 (32) | 31 (62) | 19 (38) | *NR* | *NR* |
|  |  | 5FU-Irinotecan | 53 | 0 | 39 (73.6) | 14 (26.4) | 33 (62) | 20 (38) |  |  |
| **O'Connell** [^28^](#_ENREF_28) | 2014 | 5FU | 477 | *NS* |  |  | 276 (57.9) | 201 (42.1) | *NR* | *NR* |
|  |  | 5FU-OX | 329 |  |  |  | 203 (61.7) | 126 (38.3) |  |  |
|  |  | CAP | 472 |  |  |  | 271 (57.4) | 201 (42.6) |  |  |
|  |  | CAPOX | 330 |  |  |  | 203 (61.5) | 127 (38.5) |  |  |
| **Rodel** [^29^](#_ENREF_29) | 2015 | 5FU | 623 | 32 (5.1) | 537 (86.2) | 50 (8.0) | 159 (25.5) | 451 (72.4) | *NR* | 216 (34.7) |
|  |  | 5FU-OX | 613 | 22 (3.6) | 549 (89.6) | 41 (6.7) | 146 (23.8) | 452 (73.7) |  | 249 (40.0) |
| **Valentini** [^62^](#_ENREF_62) | 2008 | Cisplatin-5FU | 83 | 0 | 83 (100) | 0 | 27 (32.5) | 56 (67.5) | *NR* | 36 (43.4)  (<3cm) |
|  |  | Raltitrexed-OX | 81 | 0 | 81 (100) | 0 | 30 (37.0) | 51 (63.0) |  | 28 (34.6)  (<3cm) |
| **Chemoradiotherapy vs. chemoradiotherapy + targeted therapy** | | | | | | | | | | |
| **Salazar** [^23^](#_ENREF_23) | 2015 | CAP | 46 | 1 (2.2) | 38 (82.6) | 7 (15.2) | 5 (10.9) | 41 (89.1) | *NR* | 16 (34.8) |
|  |  | CAP-BEV | 44 | 1 (2.3) | 33 (75.0) | 10 (22.7) | 7 (15.9) | 37 (84.1) |  | 20 (45.5) |
| **Chemoradiotherapy vs. chemoradiotherapy (CRT) + consolidation (cons.) chemotherapy** | | | | | | | | | | |
| **Kim** [^39^](#_ENREF_39) | 2018 | CAP | 55 | 0 | 45 (81.8) | 10 (18.2) | 4 (7.3) | 51 (92.7) | 16 (29.1) | 10 (18.2) |
|  |  | CRT: CAP  Cons.: CAPOX | 53 | 0 | 44 (83.0) | 9 (17.0) | 3 (5.7) | 49 (92.5) | 14 (26.4) | 9 (17.0) |
| **Moore** [^40^](#_ENREF_40) | 2017 | 5FU | 24 | 1 (4.2) | 18 (75.0) | 5 (20.8) | 2 (8.3) | 22 (91.7) | 12 (50.0) | 5 (20.8) |
|  |  | CRT: 5FU  Cons.: 5FU | 25 | 0 | 24 (96.0) | 1 (4.0) | 0 | 25 (100) | 15 (60.0) | 1 (4.0) |
| **Chemoradiotherapy vs. induction (ind.) chemotherapy + chemoradiotherapy (CRT)** | | | | | | | | | | |
| **Borg** [^33^](#_ENREF_33) | 2014 | BEV-5FU | 45 | 0 | 45 (100) | 0 | 8 (17.8) | 37 (82.2) | *NR* | *NR* |
|  |  | Ind.: BEV-FOLFOX4  CRT: BEV-5FU | 46 | 0 | 46 (100) | 0 | 10 (21.7) | 36 (78.3) |  |  |
| **Fernandez-Martos** [^34^](#_ENREF_34) | 2015 | CAPOX | 52 | 0 | 49 (94.2) | 3 (5.8) | *NR* |  | 5 (9.6) | 12 (23.1)  (≤6 cm) |
|  |  | Ind.: CAPOX  CRT: CAPOX | 56 | 0 | 49 (87.5) | 7 (13.5) |  |  | 0 (0) | 18 (32.1)  (≤6 cm) |
| **Marechal** [^35^](#_ENREF_35) | 2012 | 5FU | 29 | 3 (10.3) | 23 (79.3) | 3 (10.3) | 4 (13.8) | 25 (86.2) | 9 (31.0) | 13 (44.8) |
|  |  | Ind.: mFOLFOX6  CRT: 5FU | 28 | 1 (3.6) | 25 (89.3) | 2 (7.1) | 2 (7.1) | 26 (92.9) | 13 (46.4) | 11 (39.3) |
| **Rouanet** [^37^](#_ENREF_37) | 2017 | FOLFIRINOX | 11 | 0 | 11 (100) | 0 | 2 (18.2) | 9 (81.8) | 8 (72.7) | *NR* |
|  |  | Ind.: FOLFIRINOX  CRT: CAP | 19 | 0 | 19 (100) | 0 | 4 (21.0) | 14 (73.7) | 18 (94.7) |  |
|  |  | Ind. FOLFIRINOX  CRT: CAP | 52 | 0 | 40 (76.9) | 12 (23.1) | 2 (3.8) | 50 (96.2) | 47 (90.4) |  |
|  |  | Ind. FOLFIRINOX  CRT: CAP | 51 | 0 | 38 (74.5) | 13 (25.5) | 1 (2.0) | 49 (98.0) | 46 (90.2) |  |
| **Fokas** [^38^](#_ENREF_38) | 2019 | Ind.: 5FU-OX  CRT: 5FU-OX | 156 | 6 (3.8) | 132 (84.6) | 18 (11.5) | 16 (10.2) | 134 (85.9) | 48 (30.8) | 64 (41.0) |
|  |  | CRT: 5FU-OX  Cons.: 5FU-OX | 150 | 4 (2.7) | 118 (78.7) | 27 (18.0) | 14 (9.3) | 135 (90.0) | 33 (22.0) | 62 (41.3) |
| **SCRT-delay** | | | | | | | | | | |
| **Latkauskas** [^24^](#_ENREF_24) | 2016 | SCRTds | 68 | *NS* |  |  | 16 (23.5) | 52 (76.5) | *NR* | 34 (50.0) |
|  |  | 5FU | 72 |  |  |  | 15 (20.8) | 57 (79.2) | - | 30 (41.7) |

*Abbreviations: 5FU = 5-Fluorouracil, BEV = Bevacizumab, C = Cetuximab, CAP = Capecitabine, CAPOX = Capecitabine + Oxaliplatin, cN = clinical nodal stage, Cons. = consolidation chemotherapy, CRT= chemoradiotherapy, cT = clinical tumor stage, Ind.= Induction chemotherapy, MRF = mesorectal fascia, NR = not reported, OX = Oxaliplatin, S1 = tegafur/gimeracil/oteracil, SCRT = short-course radiotherapy*

**Supplementary Table 4.** Overview of administered therapies and dose.

| **Author** | **Neoadjuvant chemotherapy** | **Neoadjuvant radiotherapy total dose (Gy) + boost**  **(number of fractions * fraction dose)** | **Adjuvant treatment** |
| --- | --- | --- | --- |
| **Deng** | 5 cycles Lv 400 mg/m^2^  5FU 400 mg/m^2^ and 5FU 2.4 g/m^2^ in 48h | 46-50.4 Gy (23-28 * 1.8-2) | 5FU-Lv x 7 cycles |
|  | 5 cycles mFOLFOX6:  Lv 400 mg/m^2^, 5FU 400 mg/m^2^, 5FU 2.4 g/m^2^ in 48h and Oxaliplatin 85 mg/m^2^ on day 1 | 46-50.4 Gy (23-28 * 1.8-2) | mFOLFOX6 × 7 cycles |
|  | 4-6 cycles mFOLFOX6 | Before or after surgery at physician discretion | mFOLFOX6 x 6–8 cycles |
| **Gerard** | Capecitabine 800 mg/m^2^ b.i.d. 5 days/week during radiotherapy | 45 Gy (25 * 1.8) | decision left to institution |
|  | Capecitabine 800 mg/m^2^ b.i.d. 5 days/week during radiotherapy  5 injections Oxaliplatin 50 mg/m2 once weekly | 50 Gy (25 * 2) |  |
| **Jiao** | Capecitabine 800 mg/m2 b.i.d., day 1−14 and day 22−25 | 50 Gy (25 * 2) | FOLFOX x 6−8 cycles |
|  | Capecitabine 800 mg/m2 b.i.d., day 1−14 and day 22−25  Oxaliplatin 60 mg/m2, i.v. over 2 h, on day 1, 8, 22 and 29 |  |  |
| **Jung** | 2 cycles 5FU 400 mg/m2/day  Lv 20 mg/m2/day for 3 days | 45-50.4 Gy + 4.5-9.0 Gy  (25-28 * 1.8) | 5FU-LV x 4 cycles |
|  | Irinotecan 40 mg/m2 on days 1, 8,15, 22, and 29  S-1 70 mg/m2 concurrently with radiotherapy |  |  |
| **Mohiuddin** | 5FU 225 mg/m2 per day, 7 days per week | 45.6 Gy + 9.6 Gy for cT3 / 14.4 Gy for cT4)  (19 * 1.2 b.i.d.) | recommended for patients  with pathological residual disease |
|  | 5FU 225 mg/m2 per day, 5 days per week  Irinotecan 50 mg/m2 once weekly |  |  |
| **O'Connell** | 5FU 225 mg/m2/day | 45 Gy + 5.4 Gy for cT3 / 10.8 Gy for cT4 (25 * 1.8) | decision left to institution |
|  | 5FU 225 mg/m2/day  Oxaliplatin 50 mg/m2 once weekly |  |  |
|  | Capecitabine 825 mg/m2 b.i.d. |  |  |
|  | Capecitabine 825 mg/m2 b.i.d.  Oxaliplatin 50 mg/m2 once weekly |  |  |
| **Rodel** | 5FU 1000 mg/m² on days 1–5 and 29–33 of radiotherapy | 50.4 Gy (28 * 1.8) | 5FU x 4 cycles |
|  | 5FU 1000 mg/m² on days 1–5 and 29–33 & 5FU 250 mg/m² on days 1–14 and 22–35  Oxaliplatin 50mg/m² on days 1, 8, 22, and 29 |  | 5FU-OX x 8 cycles |
| **Valentini** | Cisplatin 60 mg/m2 on day 1 and 29  5FU 1,000 mg/m2 on days 1–4and 29–32 | 50.4 Gy (25 * 1.8 + 5.4) | recommended for ypN+ patients, regimen depended on physician preference |
|  | Raltitrexed 3 mg/m2  Oxaliplatin 130 mg/m2 on days 1, 19, and 38 |  |  |
| **Salazar** | Capecitabine825 mg/m^2^ b.i.d. 5 days/week during RT | 45 Gy (25 * 1.8) | administered  at the investigators discretion |
|  | Capecitabine 825 mg/m^2^ b.i.d.5 days/week during RT  & Bevacizumab 5 mg/kg on day 1, 15 and 29 |  |  |
| **Kim** | Capecitabine 825 mg/m^2^ b.i.d. 5 days/week during RT | 50.4 Gy (28 * 1.8) | ypStage 0-1: CAP x 6 cycles  ypStage II-III:  CAP+OX x 6 cycles |
|  | Capecitabine 825 mg/m^2^ b.i.d. 5 days/week during RT  Consolidation:  2 cycles Capecitabine 850 mg/m2 b.i.d.  Oxaliplatin 100 mg/m2 on day 1 |  |  |
| **Moore** | 5FU 225 mg/m2/day | 45 Gy + 5.4Gy (25 * 1.8) |  |
|  | 5FU 225 mg/m2/day  Consolidation:  3 cycles 5FU 450 mg/m2 + Lv 50 mg/m2 |  |  |
| **Borg** | Bevacizumab  5 mg/kg 2 weeks before CRT + at cycle 1, 3 and 5  & 5FU 225 mg/m2/day | 45 Gy (25 * 1.8) | left to the investigators  discretion |
|  | Induction:  6 cycles Bevacizumab 5 mg/kg  FOLFOX4 Oxaliplatin 85 mg/m2, Lv 200 mg/m2, 5FU bolus 400 mg/m2, + i.v. 600 mg/m2  CRT:  Bevacizumab 5 mg/kg 2 weeks before CRT and at cycle 1, 3 and 5  5FU 225 mg/m2/day |  |  |
| **Dewdney** | Induction:  4 cycles Capecitabine 850 mg/m2 b.i.d. for 2 weeks  Oxaliplatin 130mg/m2 on day 1  CRT:  Capecitabine 825 mg/m2 b.i.d. | 45Gy + 5.4Gy (25 * 1.8) | CAP-OX  x 4 cycles |
|  | Induction:  4 cycles Capecitabine 850 mg/m2 b.i.d. for 2 weeks  Oxaliplatin 130mg/m2 on day 1  Cetuximab 250mg/m2/wk  CRT:  Capecitabine 825 mg/m2 b.i.d  Cetuximab 250 mg/m2 weekly |  |  |
| **Fernandez-Martos** | Capecitabine 825 mg/m2 b.i.d.  Oxaliplatin 50 mg/m2/day on days 1, 8, 15, 22, and 29 | 50.4 Gy (28 * 1.8) | CAPOX x 4 cycles |
|  | Induction  4 cycles Capecitabine 2,000 mg/m2 for 14 days  Oxaliplatin 130 mg/m2 on day 1 of each cycles  CRT:  Capecitabine 825 mg/m2 b.i.d.  Oxaliplatin 50 mg/m2/day on days 1, 8, 15, 22, and 29 |  | - |
| **Marechal** | 5FU 225 mg/m2/day | 45 Gy (25 * 1.8) |  |
|  | Induction:  2 cycles mFOLFOX6:  Oxaliplatin 100 mg/m2 i.v. in 2 h on day 1,  folinic acid 400 mg/m2 on day 1,  5FU bolus 400 mg/m2 on day 1 + 5FU 2000 mg/m2 i.v. in 46 h  CRT:  5FU 225 mg/m2/day |  |  |
| **Rouanet** | 4 cycles FOLFIRINOX:  Irinotecan 180 mg/m2  Oxaliplatin 85 mg/m2  Elvorin 200 mg/m2  5FU 400 mg/m2 bolus + 2400 mg/m2 in 46h | None | left to the investigators  discretion. Advise:  ypT0-1N0 no adjuvant treatment.  ypT ≥ 2 or ypN ≥ 1: FOLFOX  x 6 cycles cycles |
|  | Induction:  FOLFIRINOX  CRT:  Capecitabine 800 mg/m2 b.i.d. | 50 Gy (25 * 2) |  |
|  | Induction:  FOLFIRINOX  CRT:  Capecitabine 800 mg/m2 b.i.d | 50 Gy (25 * 2) |  |
|  | Induction:  FOLFIRINOX  CRT:  Capecitabine 800 mg/m2 b.i.d | 60 Gy (30 * 2) |  |
| **Fokas** | Induction:  3 cycles Oxaliplatin 100 mg/m2, Lv 400 mg/m2, 5FU 2,400 mg/m2  CRT:  5FU 250 mg/m2 on days 1-14 and 22-35  Oxaliplatin 50 mg/m2 on days 1, 8, 22, and 29 | 50.4 Gy (28 * 1.8) | Not recommended |
|  | CRT:  5FU 250 mg/m2 on days 1-14 and 22-35  Oxaliplatin 50 mg/m2 on days 1, 8, 22, and 29  Consolidation:  3 cycles Oxaliplatin 100 mg/m2  Lv 400 mg/m2  5FU 2,400 mg/m2 |  |  |
| **Latkauskas** | None | 25 Gy (5 * 5) | 5FU-LV  x 4 cycles |
|  | 5FU 400 mg⁄m2  Lv 20 mg ⁄m2 | 50 Gy (25 * 2) |  |
